# Clinical diagnosis model of spinal meningiomas based on the surveillance, epidemiology, and end results database

**DOI:** 10.3389/fsurg.2023.1008605

**Published:** 2023-02-14

**Authors:** Yong’An Jiang, Peng Chen, JiaWei Liang, XiaoYan Long, JiaHong Cai, Yi Zhang, ShiQi Cheng, Yan Zhang

**Affiliations:** ^1^ Department of Neurosurgery, The Second Affiliated Hospital of Nanchang University, Nanchang, China; ^2^ Nanchang University, Nanchang, China; ^3^ East China Institute of Digital Medicine, Shangrao, China

**Keywords:** spinal meningiomas, SEER database, nomogram, prognostic, LASSO

## Abstract

Most spinal meningiomas (SM) are benign lesions of the thoracic spine and are usually treated surgically. This study aimed to explore treatment strategies and construct a nomogram for SM. Data on patients with SM from 2000 to 2019 were extracted from the Surveillance, Epidemiology, and End Results database. First, the distributional properties and characteristics of the patients were descriptively evaluated, and the patients were randomly divided into training and testing groups in a 6:4 ratio. Least absolute shrinkage and selection operator (LASSO) regression was used to screen the survival predictors. Kaplan–Meier curves explained survival probability by different variables. The nomogram was constructed based on the results of LASSO regression. The predictive power of the nomogram was identified using the concordance index, time-receiver operating characteristics, decision curve analysis, and calibration curves. We recruited 1,148 patients with SM. LASSO results for the training group showed that sex (coefficient, 0.004), age (coefficient, 0.034), surgery (coefficient, −0.474), tumor size (coefficient, 0.008), and marital status (coefficient, 0.335) were prognostic factors. The nomogram prognostic model showed good diagnostic ability in both the training and testing groups, with a C-index of 0.726, 95% (0.679, 0.773); 0.827, 95% (0.777, 0.877). The calibration and decision curves suggested that the prognostic model had better diagnostic performance and good clinical benefit. In the training and testing groups, the time-receiver operating characteristic curve showed that SM had moderate diagnostic ability at different times, and the survival rate of the high-risk group was significantly lower than that of the low-risk group (training group: *p* = 0.0071; testing group: *p* = 0.00013). Our nomogram prognostic model may have a crucial role in predicting the six-month, one-year, and two-year survival outcomes of patients with SM and may be useful for surgical clinicians to formulate treatment plans.

## Introduction

Meningiomas are primary intracranial tumors, most of which are benign tumors of the central system, with an annual incidence of about five cases per 100,000 people (1–3). Spinal meningiomas (SMs) are very rare, accounting for only 2%–12% of meningiomas, with approximately 0.25% of male patients; it is the most common primary spinal tumor in adults, most often growing in the extradural extramedullary site, with an incidence of only 0.33 cases per 100,000 population (4–6).

The main treatment strategy for SM is surgery that avoids the compression of nerves after tumor resection and removes the tumor with little risk of complications and recurrence (7, 8). The choice of surgical approach (complete resection) remains controversial. Moreover, poor outcomes for SM have been reported to be related to age, sex, and tumor size (9–12). Accurate prediction of survival time, as well as prognostic factors of SM, is crucial, so new treatment strategies need to be developed to improve patient survival.

The Surveillance, Epidemiology, and End Results (SEER) database includes a large sample of the United States population data (13, 14), which undoubtedly increases the reliability compared to a single-center study with a small sample. This study aimed to explore the prognostic factors of SM and to identify the best treatment strategy.

## Materials and methods

### Study data collection

According to the National Cancer Institute, the SEER database includes demographic and survival data for more than 28% of cancer patients in the United States. This retrospective analysis included patients of SM with a histologically confirmed diagnosis between 2000 and 2019 (*n* = 1148). Patients diagnosed with meningioma in the spinal cord (C72.0- spinal cord, C70.1- spinal meninges, and C72.1- cauda equina) were included. The term meningiomas was recruited by setting the variable “Histology recode - broad groupings” as “9,530–9,539: meningiomas”. The clinical information extracted of the patients with SM included: age, sex, race, laterality, surgery, tumor size, overall survival (OS) time, OS status, and marital status. The exclusion criteria were (1) patients with a survival time of zero or unknown, (2) patients without histologically confirmed positive SM, and (3) patients who underwent an unknown surgical method.

### Study design

**Figure 1:**
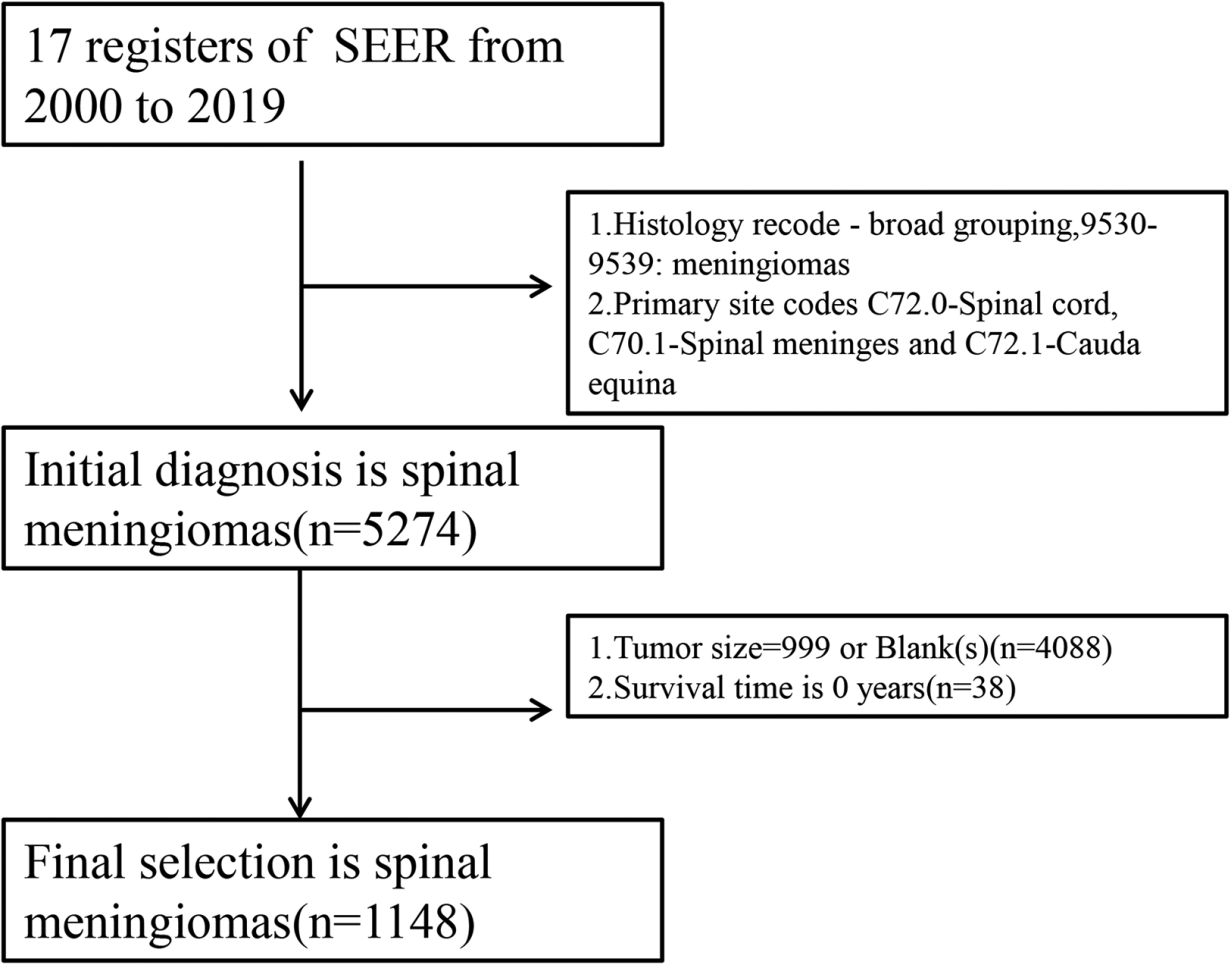
Flowchart of the analysis of spinal meningiomas.

In this study, after filtering the extracted data, the clinical data of the patients with SM were classified as follows: age group (≥81 years and <81 years, the optimal age was defined by the “survminer” package, version: 0.4.9); sex (female and male); race [Black, Other (American Indian/ Alaska Native, Asian/Pacific Islander), White and Unknown]; laterality (left, paired site or not and right); surgery (biopsy and “GTR_or_STR”); tumor size (≥26 and <26, optimal size is defined by “survminer” package); and marital status [married (including common law), others]. For surgical modalities, either gross total resection (GTR) or subtotal resection (STR) was considered to be non-biopsy. A detailed flowchart is shown in Figure 1. The statistical analysis is described in the Supplementary Table S1.

## Results

### Baseline characteristics of the patients with SM

According to the established criteria, a total of 1,148 (training group vs. testing group: 688 vs. 460, Supplementary Table S2) patients with SM were recruited for our study analysis. The median survival time was 21 months. In detail, 1,079 (94%) of the patients survived, while 69 (6%) patients died (clinical characteristics of the entire population of patients with SM can be reviewed in Table 1). The optimal cutoff values of age and tumor size were 81 years old and 26 mm, respectively. There were 144 (21%) and 544 (79%) males and females in the training group, respectively, and 90 (20%) and 370 (80%) males and females in the testing group, respectively. In the training and testing groups, 31 (5%) and 30 (7%) of the SMs, respectively, were unilateral, and 657 (95%) and 430 (93%), respectively, were bilateral.

**Table 1:** Clinical characteristics of the patients with SM (*n* = 1,148).

Characteristic	Freq
Sex, No. (%)	
Female	914 (79.6%)
Male	234 (20.4%)
Laterality, No. (%)	
Unilateral	61 (5.3%)
Bilateral	1,087 (94.7%)
Age, No. (%)	
≥81	149 (13%)
<81	999 (87%)
Surgery, No. (%)	
Biopsy	258 (22.5%)
GTR_or_STR	890 (77.5%)
Tumor_size, No. (%)	
≥26	185 (16.1%)
<26	963 (83.9%)
OS_Time, median (interquartile range)	21 (10 to 33)
OS_Status, No. (%)	
Alive	1,079 (94%)
Dead	69 (6%)
Marital_status, No. (%)	
Married	642 (55.9%)
Single	506 (44.1%)
Race, No. (%)	
White	917 (80.6%)
Black	81 (7.1%)
Others/Unknown	140 (12.3%)

SM, spinal meningioma; GTR, gross total resection; STR, subtotal resection; OS time, overall survival time.

In the training group, the surgical method was GTR or STR and biopsy in 543 (79%) and 145 (21%) patients, respectively. In the testing group, the surgical method was GTR or STR and biopsy in 347 (75%) and 113 (25%) patients, respectively. Tumor size was ≥26 mm and <26 mm in 112 (16%) and 576 (84%) patients, respectively, in the training group and 73 (16%) and 387 (84%) patients, respectively, in the testing group. In the training group, 546 (80%), 51 (7%), and 86 (13%) patients were White, Black, and Other/Unknown, respectively. In the testing group, 371 (81%), 30 (7%), and 54 (12%) patients were White, Black, and Other/Unknown, respectively. In the training group, 86 (12%) and 602 (88%) of patients were ≥81 years old and <81 years old, respectively. In the testing group, 63 (14%) and 397 (86%) of patients were ≥81 years old and <81 years old, respectively. In the training and testing groups, 373 (54%) and 269 (58%) of patients were married, respectively, and 315 (46%) and 191 (42%) were single. There was no statistical difference between the training and testing groups (*p* > 0.05) (Supplementary Table S1).

### Least absolute shrinkage and selection operator (LASSO) regression in the training group

**Figure 2:**
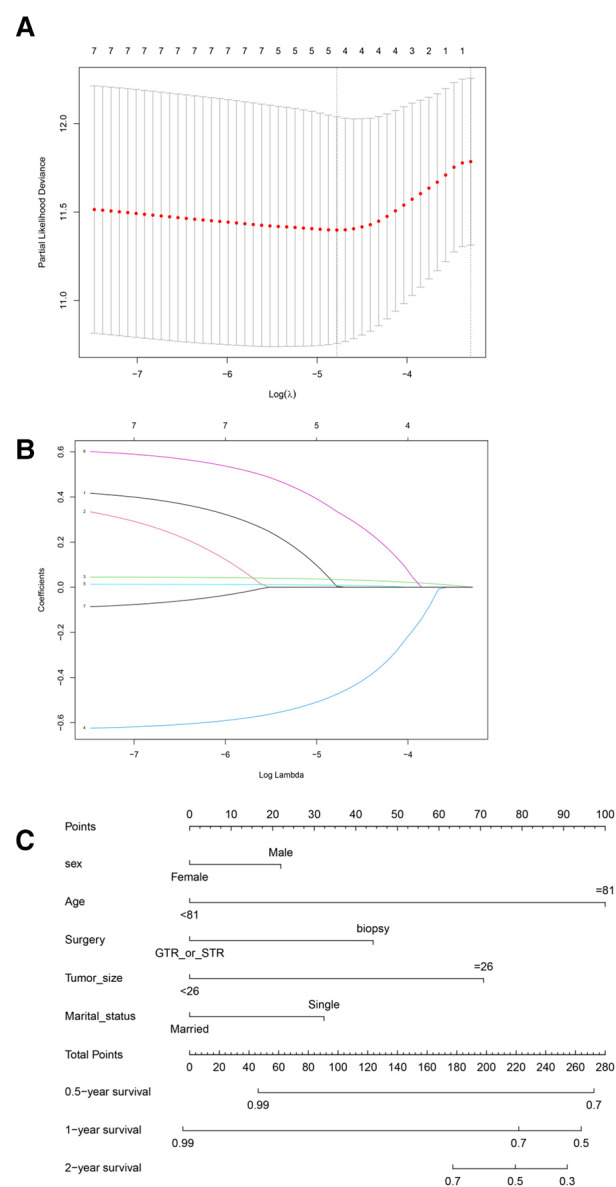
Least absolute shrinkage and selection operator (LASSO) regression analysis. (**A**) LASSO regression parameter characteristics (regression coefficient is not equal to 0); (**B**) Select LASSO parameters (*λ*). (**C**) Prognostic nomogram constructed according to five risk factors of spinal meningioma in six months, one year, and two years. GTR or STR, gross total resection or subtotal resection.

All SM-related variables were screened by LASSO regression: sex (coefficient, 0.004), age (coefficient, 0.034), surgery (coefficient, −0.474), tumor size (coefficient, 0.008), and marital status (coefficient, 0.335). These are shown in Figure 2, with the dark blue line No. 4 (surgery), purple line No. 6 (marital status), black line No. 1 (sex), green line No. 3 (age), and light blue line No. 5 (tumor size). We applied five prognostic variables based on the minimum Lambda value.

### Prognostic power of the nomogram

We constructed a nomogram based on the prognostic variables screened by the above LASSO regression and explored the results through the proportional hazards hypothesis: sex (*p* = 0.129), age (*p* = 0.0000539), surgery (*p* = 0.055), tumor size (*p* = 0.089) and marital status (*p* = 0.001). The six-month, one-year, and two-year survival probability scores of SM were based on the above five clinical features (Figure 2C).

### Assessing the accuracy of the nomogram

**Figure 3:**
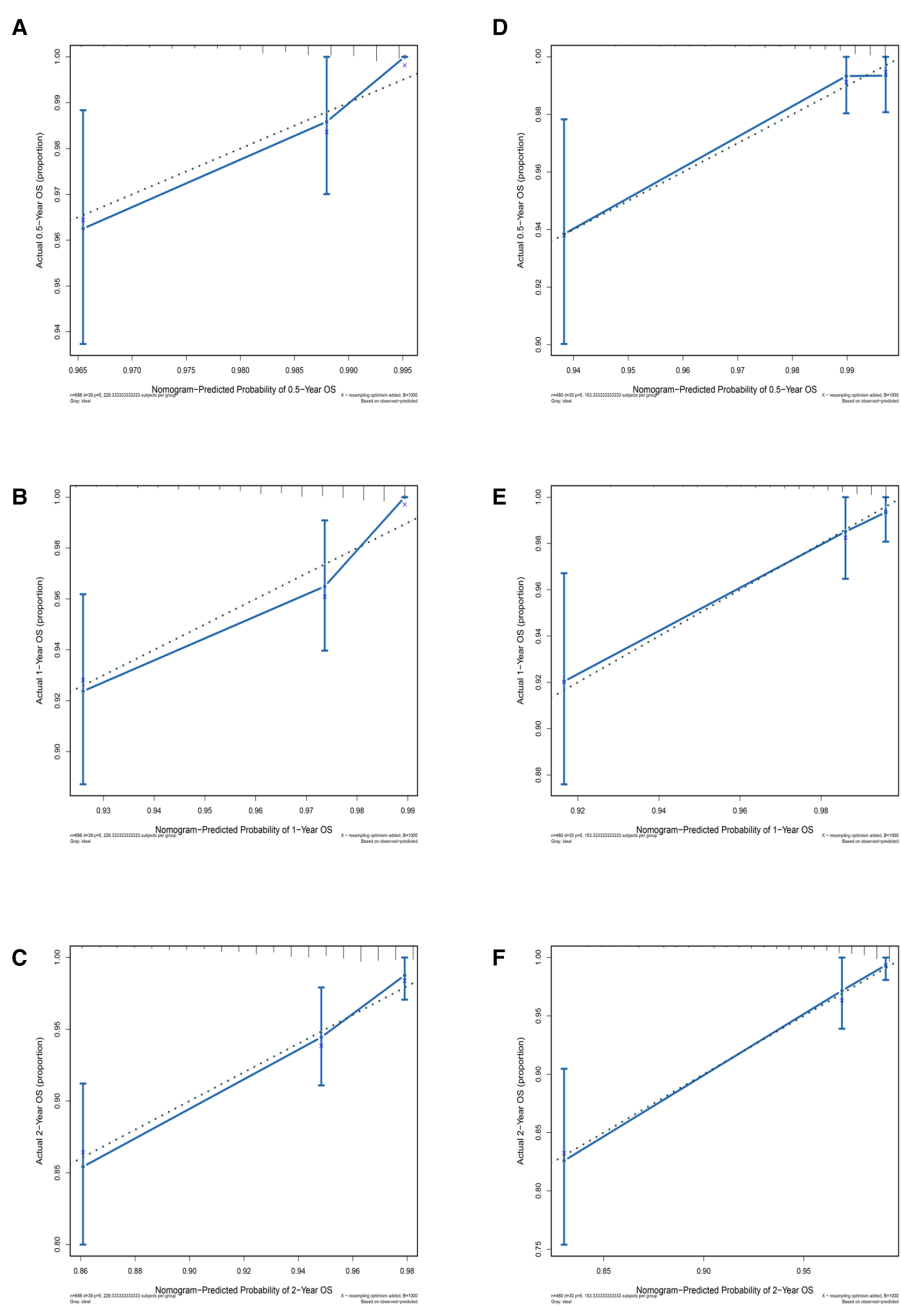
Calibration curves for different years of training and testing prognostic nomograms. Calibration curve for the training group at six months, one year, and two years. (**D–F**) Calibration curve for the testing group at six months, one year, and two years.

We used the population characteristics of the training group and the testing group to obtain a C-index of 0.726, 95% (0.679, 0.773); 0.827, 95% (0.777, 0.877). The calibration curve showed that the training and test groups had good agreement between the six-month, one-year, and two-year predictions and actual observations in patients with SM (Figure 3; left, training group; right, testing group).

### Decision curve analysis (DCA) to evaluate clinical models

**Figure 4:**
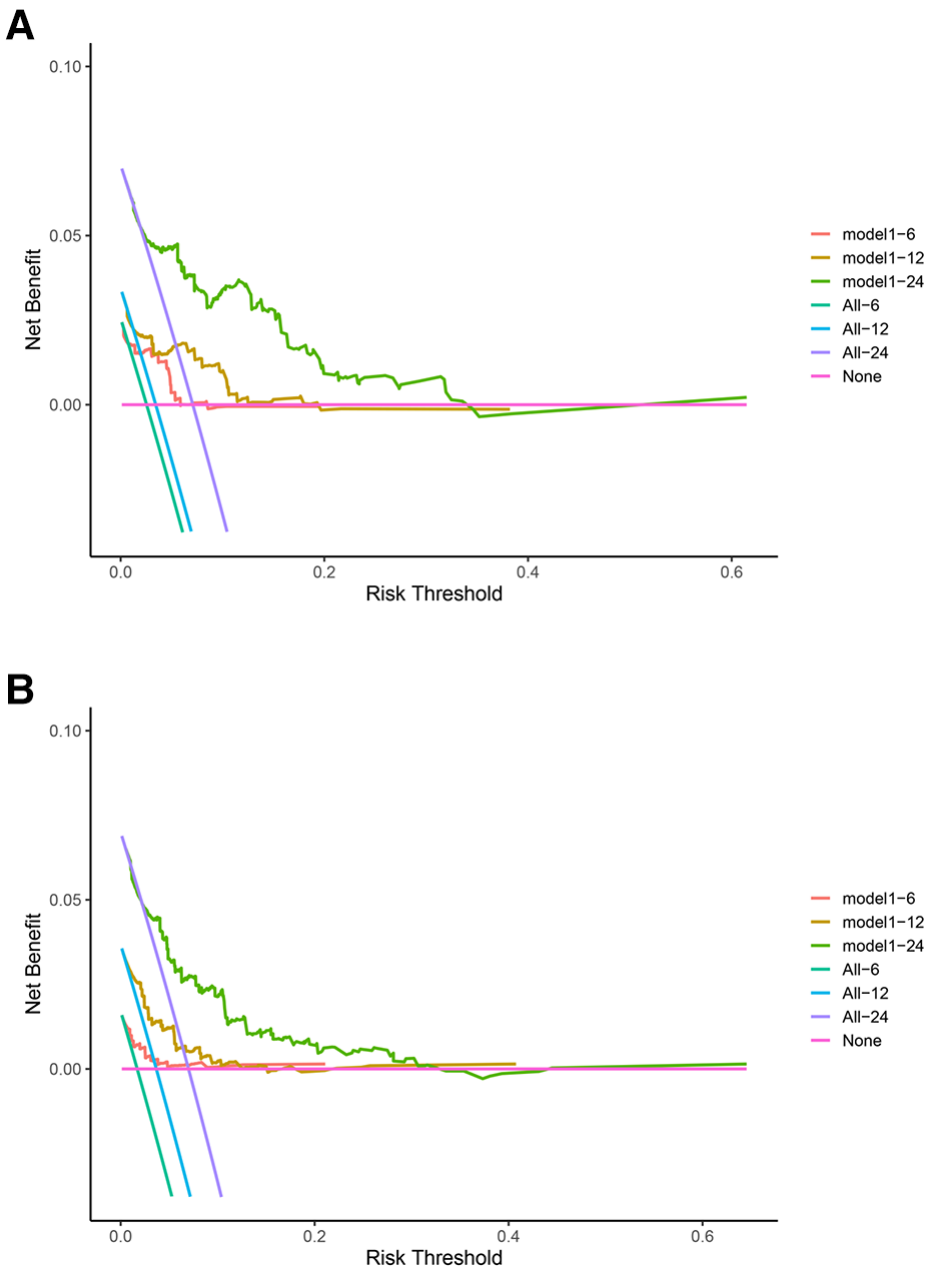
Decision curve analysis (DCA) for the spinal meningioma model. (**A**) DCA of the training group for six months, one year, and two years. (**B**) DCA of the testing group for six months, one year, and two years. The horizontal axis refers to the threshold probability situation, and the vertical axis represents the net benefit change.

DCA was used to assess the SM clinical model constructed by the nomogram (Figure 4). DCA was used to evaluate the SM clinical model constructed by the nomogram, both in the training and test groups, indicating that the nomogram model had clinical benefits for estimating the prognosis of SM.

### Time-dependent receiver operating characteristic curve

**Figure 5:**
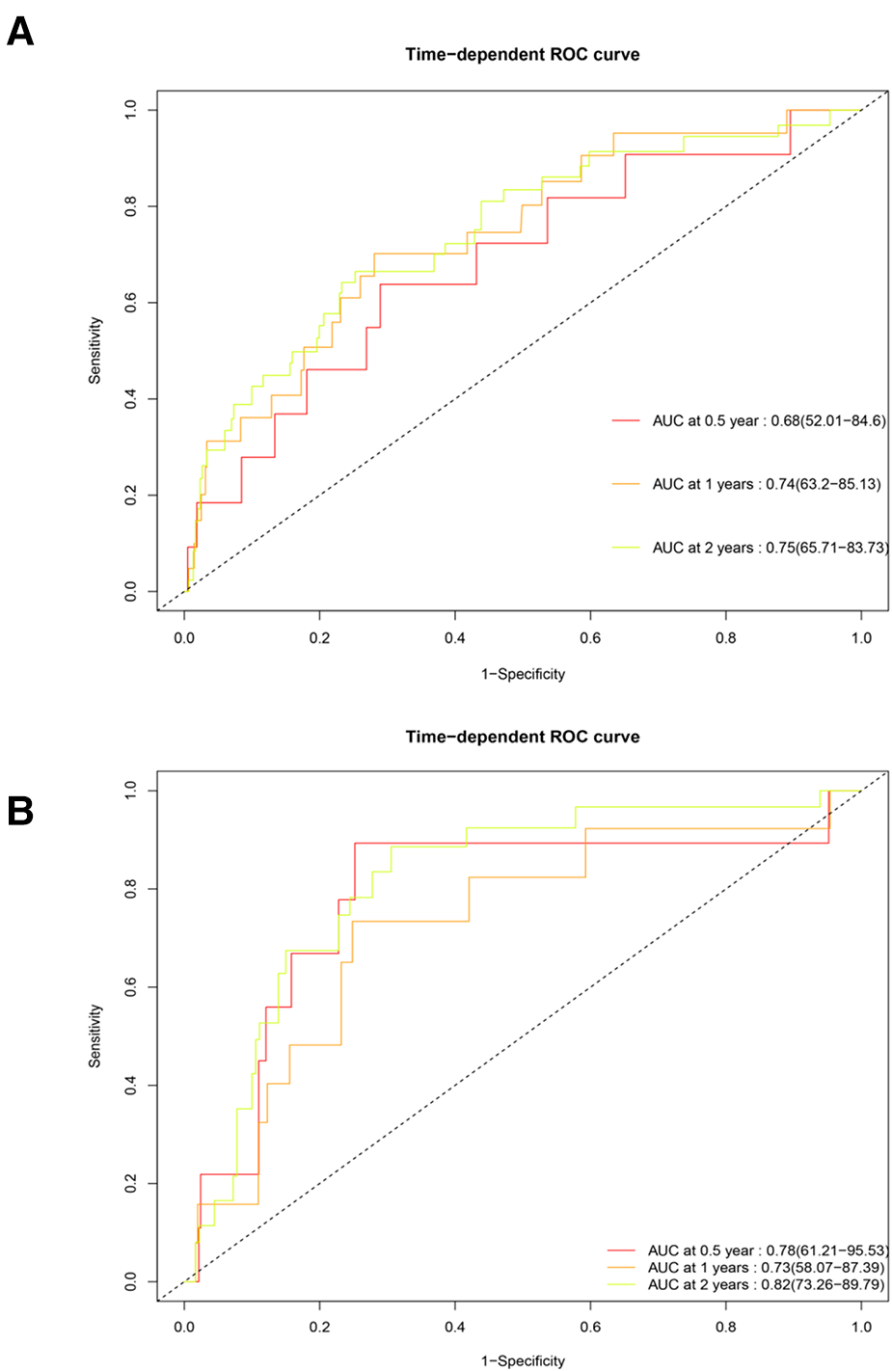
Time-dependent receiver operating characteristic curve of the training and testing groups. (**A**) Training group. (**B**) Testing group. The horizontal axis represents specificity, and the vertical axis represents sensitivity. AUC, area under the curve.

The Cox proportional hazards regression model was used to estimate the survival of patients with SM at different times, and this process relied on the “timeROC” package (Figure 5). In the training group, the area under the curve (AUC) of the model for six months was 0.68, the AUC for one year was 0.74, and the AUC for two years was 0.75. In the testing group, the AUCs for six months, one year, and two years were 0.79, 0.73, and 0.82, respectively. All of the above indicate that the model had fairly good accuracy.

### Survival analysis results of the model

**Figure 6:**
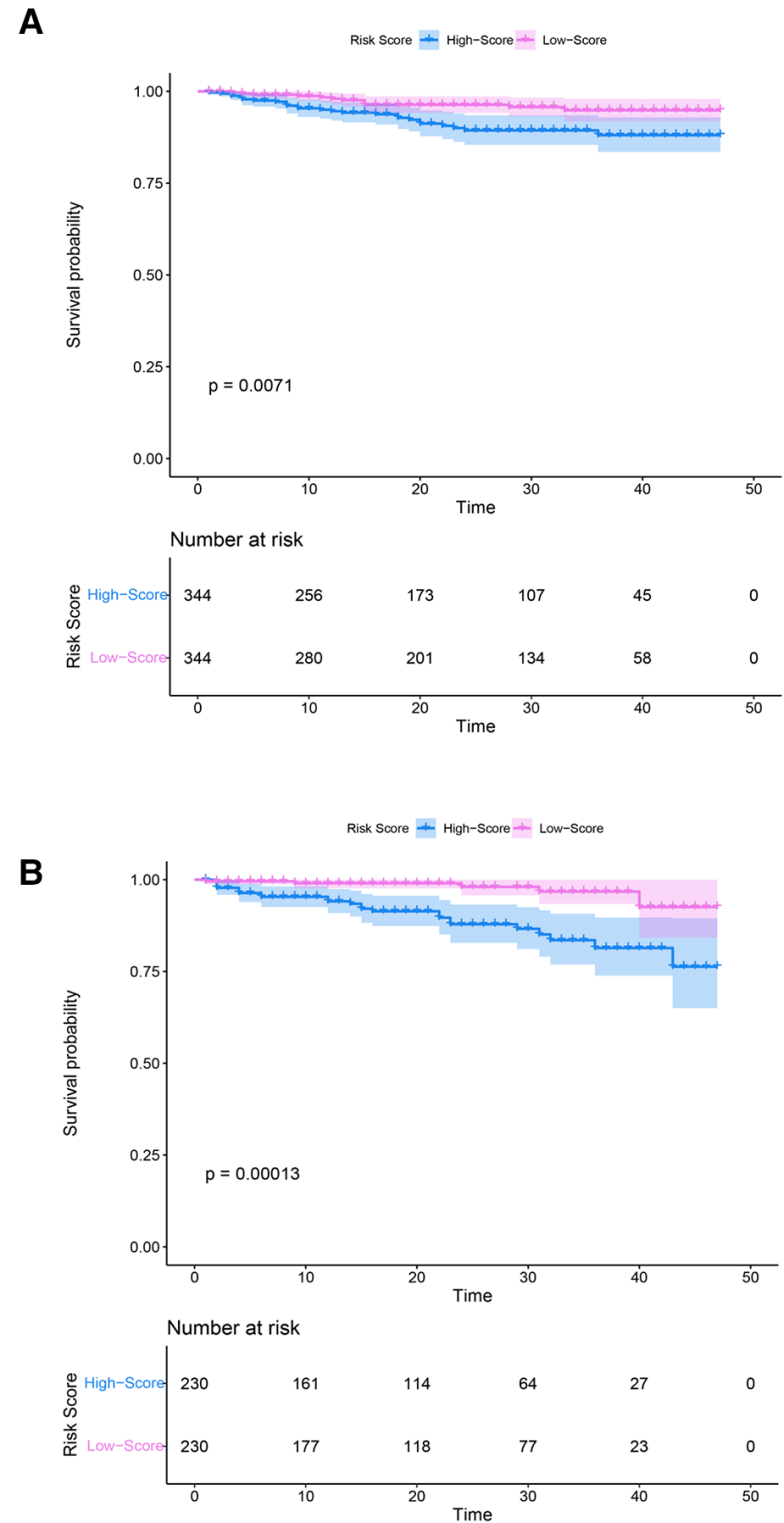
Kaplan–Meier curves of the training and testing groups. (**A**) Training group. (**B**) Testing group. Purple and blue represent confidence intervals. The horizontal axis represents time (months), and the vertical axis represents survival probability.

The risk score was constructed according to the coefficients (coef) of all variables obtained from the Cox proportional hazards regression model; the ones above the median were defined as the high-score group, and those below the median were the low-score groups. The survival curve results of patients with SM in the training (*p* = 0.0071) and testing groups (*p* = 0.00013) showed that the survival rate of the high-score model group was lower than that of the low-score model group (Figure 6).

## Discussion

SM are the most common benign spinal tumors in adults, accounting for 25%–45% of all tumors, with an age-adjusted incidence of 0.33 cases per 100,000 population (4–6, 12). In this study, we obtained patient data of SM from a very large population clinical sample (SEER) and constructed a clinical prediction nomogram model of the six-month, one-year, and two-year survival probability. Our results suggest that sex, age, surgery, tumor size, and marital status are important predictors of prognosis in patients with SM. Furthermore, through the multi-faceted evaluation of the nomogram, it was confirmed that the SM nomogram model had good clinical applicability and could be used as a clinical prediction model for SM. To the best of our knowledge, this study is the largest multicenter retrospective study with a more accurate interpretation and broader clinical application for SM.

As confirmed by previous studies, SM occurs mainly in the elderly, between the ages of 70–90 years, and is more common in female patients, with a male:female ratio of approximately 1:4 (15). Of the patients included in our study, 79.6% were female and 20.4% male. In addition, according to the “survminer” package for all samples, the optimal age was 81 years, which is consistent with previous studies. Westwick (9) also showed that patients with SM over the age of 80 had the lowest all-cause survival, with a lower incidence in males. Cao (8) found the male-to-female ratio to be 4:1 and the OS rate of female patients to be higher than that for males. Regarding the surgical approach, many studies generally agree that improving neurological outcomes is the best choice for treating SM, with low recurrence after resection and that Simpson class I resection should be performed when possible (16–18). Wu et al. (19) concluded that GTR was achieved in 94.5% of SM cases (Simpson I and II), and STR (Simpson III or higher) was achieved in 5.5% of cases. Both GTR and STR were preferred as treatments for SM. In our study, the surgical modality (GTR and STR) was considered an important prognostic factor affecting patient survival, which inspired us, when faced with treatment options for patients with SM, to try to remove as many tumor cells as possible and stop the progression of the disease.

We constructed a novel nomogram from SEER clinical data with a high C-index and better calibration curve results. We speculate that the five prognostic factors screened by LASSO can reflect the prognosis and survival of patients with SM. In addition, the clinical benefit curve (decision curve) reflects the better feasibility of the clinical prediction model. Therefore, our nomogram could help surgeons to predict the six-month, one-year, and two-year survival probability of patients with SM and provide a reference for disease treatment.

Of course, we acknowledge that this study also has several limitations. First, the information we obtained from the SEER database is observational and may have been affected by selection bias. Furthermore, patients with missing information may have been excluded during the data screening stage, which could have led to further selection bias. Second, this study is retrospective, and despite its sizable sample size, a prospective study is needed to comprehensively evaluate patients with SM. Third, some important test data, such as information on lifestyle and imaging findings, are not included in SEER data. Nevertheless, this study has the characteristics of a multicenter, large-sample study and can help to provide a new clinical solution for the treatment of SM.

SM is considered to be a multifactorial disease in clinical practice. In the continuous exploration process of clinicians and researchers, more clinical factors are considered the key to prognosis. Due to the long development time of SM and the lack of related research, the formulation of the prognosis of patients with this type of disease is often incomplete. Based on public large-scale population sample data, our research comprehensively analyzes the clinical data and disease characteristics of SM and constructs a prognostic model with good diagnostic ability, which can be used to help clinicians formulate tailor-made treatment plans for patients.

In conclusion, we developed and validated a nomogram for predicting survival at six months, one year, and two years in patients with SM. This nomogram has good predictive power and could help to develop personalized treatment plans for patients with SM.

## Data Availability

The raw data supporting the conclusions of this article will be made available by the authors, without undue reservation.
