# Supplementary material for: Clinical diagnosis model of spinal meningiomas based on the surveillance, epidemiology, and end results database
Source: Front Surg. 2023 Feb 14;10:1008605. doi: 10.3389/fsurg.2023.1008605 (PMC9971498; doi:10.3389/fsurg.2023.1008605)
Supplement: Supplementary file 1 [file Table1.pdf]

Supplementary Material

1 Supplementary Tables

Table S1: Statistical analysis

Statistical analysis

All statistical analysis results in this study were generated in the R environment (R version 4.1.3, https://www.r-project.org). All SM patients were randomly divided into a training group and a testing group according to the "sample" basic package 6:4 ratio, and the chi-square test (n>5) and the continuity-corrected chi-square test (n≥1, n<5) were used to compare the two groups. Limit feature between groups. Least Absolute Shrinkage and Selection Operator (LASSO) regression was used to select the best predictors of 0.5-year, 1-year and 2-year survival in training group, and the smallest lambda value was used for variable screening. Based on the LASSO screening results, the cox proportional hazards model was constructed, and the risk score was calculated according to the formula, Risk Score = β1X1 + β2X2 + ... + βnXn (β, regression coefficient; X, prognostic factor)[1], and the median risk score was used to divide SM patients into a High Score and Low Score group. A nomogram was constructed from the LASSO results to predict the survival rate of SM at 0.5-, 1-, and 2-years. The performance of the nomogram model was evaluated by the consistency index (C-index). The calibration curve assesses the consistency of the nomogram. Decision curve analysis (DCA) was used to assess the benefit of the model for the clinical diagnosis of SM. The Kaplan-Meier (KM) curve and the time-dependent receiver operating characteristic curve (Time-dependent ROC) are both based on the Cox proportional hazards regression model. In addition, continuous variables are expressed as median (interquartile range), and categorical variables are expressed as percentages (%). $p<0.05$ was considered statistically significant.

1 Zhang, M. *et al.* An Immune-Related Signature Predicts Survival in Patients With Lung Adenocarcinoma. *Front Oncol* **9**, 1314, doi:10.3389/fonc.2019.01314 (2019).

Table S2: Clinical characteristics of the training and testing groups.

Variables	Total (n = 1148)	Testing Group(n = 460)	Training Group(n = 688)	*p*
sex, n (%)				0.626
Female	914 (80)	370 (80)	544 (79)	
Male	234 (20)	90 (20)	144 (21)	
Laterality, n (%)				0.174
Unilateral	61 (5)	30 (7)	31 (5)	
Bilateral	1087 (95)	430 (93)	657 (95)	
Age, n (%)				0.616
≥81	149 (13)	63 (14)	86 (12)	
<81	999 (87)	397 (86)	602 (88)	
Surgery, n (%)				0.188

Biopsy	258 (22)	113 (25)	145 (21)	
GTR_or_STR	890 (78)	347 (75)	543 (79)	
Tumor_size, n (%)				0.918
≥26	185 (16)	73 (16)	112 (16)	
<26	963 (84)	387 (84)	576 (84)	
Marital_status, n (%)				0.172
Married	642 (56)	269 (58)	373 (54)	
Single	506 (44)	191 (42)	315 (46)	
Race, n (%)				0.781
White	917 (81)	371 (81)	546 (80)	
Black	81 (7)	30 (7)	51 (7)	
Others/Unknown	140 (12)	54 (12)	86 (13)	

GTR, gross total resection; STR, subtotal resection.
